# The impact of surgical intervention on the psychosocial health and quality of life of children with strabismus

**DOI:** 10.3389/fpsyg.2026.1653233

**Published:** 2026-03-11

**Authors:** Ruiheng Wang, Weiling Gou, Fang Liu, Yang Zhang

**Affiliations:** 1 Department of Psychiatry, The First Affiliated Hospital of Kunming Medical University, Kunming, Yunnan, China; 2 Department of Ophthalmology, The First Affiliated Hospital of Kunming Medical University, Kunming, Yunnan, China

**Keywords:** children, discrimination, quality of life, self-esteem, social anxiety, strabismus, surgery

## Abstract

**Objective:**

To compare the psychosocial health and quality of life of children with different types and prism diopters (Δ) of strabismus, and to observe the impact of surgery on the psychosocial health and quality of life of children with strabismus.

**Methods:**

We use questionnaires to evaluate the scores of perceived discrimination (PD), social anxiety (SAD), self-esteem (S-E), and quality of life (QoL) of children. These indicators are compared between children with and without strabismus, as well as changes before and after strabismus surgery.

**Results:**

1. Children with strabismus had lower QoL and S-E scores than those without strabismus, while their SAD and PD scores were higher (*p* < 0.05). 2. Children with intermittent exotropia had higher QoL scores than the other types of strabismus (*p* < 0.05), vertical strabismus had higher SAD scores than the other three types (*p* < 0.05); 3. Patients with exotropia (-15Δ ≤ ∼ < -30Δ) had higher sores on QoL and S-E, had lower scores on SAD and individual PD than those with exotropia ( ≤ -30Δ) (*p* < 0.05). Patients with esotropia (+10Δ ≤ ∼ < +30Δ) had higher scores on QoL and S-E, had lower scores on group PD than those with esotropia ( ≥ +30Δ) (*p* < 0.05); 4. There were no significant differences in the scores of QoL and S-E between strabismus patients pre and 1 month post-surgery. These scores significantly increased 3 months after surgery (*p* = 0.000). There were no significant differences in some sub-dimensions of S-E between pre and post-surgery. There were no significant differences in SAD between pre and 1-mon-post-surgery, but it significantly decreased 3 months post-surgery compared to pre-surgery. There were some differences in PD between pre and post-surgery, although they didn’t reach the traditional statistical significance level (*p* = 0.056). Individual PD significantly decreased at 1 and 3 months after surgery (*p* = 0.010), and group PD significantly decreased at 3 months after surgery (*p* = 0.048).

**Conclusion:**

Children with strabismus have lower psychosocial health and quality of life than those without strabismus. The type and severity of strabismus is correlated with mental health and quality of life. Surgery improved S-E and QoL for strabismus patients effectively and reduced PD and SAD. However, these changes exhibit a certain latency, typically occurring 3 months after surgery.

## 1 Introduction and background

Strabismus (squint, deviation) is a kind of eye disease in children, which was influenced by various factors such as family genetic history, prematurity, intracranial lesions, trauma, ocular lesions, and refractive errors (Yetkin and Turkman, 2023; Zhang et al., 2021). The prevalence of strabismus varies significantly in the world. Hashemi et al. (2019) had conducted a meta-analysis of 56 studies from different countries and regions, which involved a total of 229,396 strabismus patients, and found the morbidity of strabismus, exotropia, and esotropia was 1.93% (95% CI:1.64–2.21), 1.23% (95% CI:1.00–1.46), and 0.77% (95% CI:0.59–0.95), respectively. A recent report from China showed the morbidity of strabismus among preschool children in eastern China was 5.56% (95% CI: 4.54–6.57%) (Wang et al., 2021), which was relatively high. During the sensitive period of visual development in children (generally considered to be under the age of 12), strabismus can lead to visual function disorders, which not only result in amblyopia or stereoscopic vision defects, but impair learning and daily living abilities. Additionally, the abnormal face appearance with strabismus may lead to discrimination, prejudice, social isolation, bullying, and other unfair treatment, which cause negative emotions and psychological issues such as low self-esteem, anxiety, loneliness, and depression, which can hinder normal psychological health development in children. When they grow up, the patients with strabismus are more likely to face challenges in employment, people relationship and their marriage, which may have negative impacts on psychosocial health and quality of life. A great deal of evidence indicated that children and adolescents with strabismus are more prone to psychological issues than those without strabismus. For example, Chinese children or adolescents aged from 10 to 17 with strabismus commonly have association with alcohol dependance as well as emotional disorders such as depression and anxiety (Chia et al., 2010). A study on strabismus and mental disorders found that children with strabismus have a higher morbidity of mental disorders (Lee et al., 2022). Similarly, Buffenn’s (2021) research concluded that persistent negative effects of strabismus elevate the risk of developing mental illness. Individuals with strabismus face challenges in self-image, employment opportunities, interpersonal relationships, sports, academics, and work (Mason et al., 2024). The social and psychological issues associated with strabismus begin in childhood and become worsen during the period of adolescence and adulthood. The most effective treatment for strabismus is surgery, which can correct ocular misalignment (improving appearance) and recovery the visual function. Recent years, there is little research on the psychosocial health and quality of life of children with strabismus in China, as well as the impact of surgical intervention on these factors. To address these deficiencies, we conducted a related survey study.

## 2 Materials and methods

This prospective study enrolled 370 children diagnosed with strabismus requiring surgical intervention from the department of ophthalmology of the two general hospitals and one specialist eye hospital during the period from June to December 2021, in Kunming City, Yunnan Province, China. These participants were allocated to the surgical intervention group (strabismus group), aged between 8 and 17 years, who met the criteria of having no intellectual or cognitive impairments, no other severe systemic diseases. Concurrently, 385 healthy children aged 8–17 years without strabismus, without other organic eye diseases, or systemic diseases were selected from primary and secondary schools in Kunming City and Zhenxiong County, Yunnan Province, China, to allocated to the control group. All participants and their guardians in this study consented to fill in the questionnaire survey. As for the other entry criteria, both the strabismus group and the control group could have concomitant refractive errors. Children in the strabismus group required a disease duration of over 6 months and could allow to have concomitant monocular or binocular amblyopia. However, the control group could not have amblyopia existed, and monocular corrected visual acuity must reach to 0.8 or above. Both groups must exclude other ophthalmic conditions such as congenital corneal disorders, cataracts, glaucoma, and retinal macular degeneration. The questionnaire was completed independently by the children, or with the guardians’ assistance. Finally, a total of 370 questionnaires were distributed to strabismus patients prior to surgery, yielding 346 valid responses (93.51%).

All surgeries involved in this study were performed by highly qualified and experienced specialists in the strabismus team, which had achieved a successful outcome rate approaching 100%. The criteria for the successful outcome are assessed primarily by the appearance correction and the remaining strabismus degree after surgeries, which specifically manifested as: For exotropia or esotropia: corneal light reflection was in the center position, with prism-lens + alternative covering test was within ± 5Δ at near distance. For vertical strabismus: corneal light reflection was also in the center position, alternative covering test was R/L or L/R, but very slightly, which prism-len-power within 3–5Δ correspondingly at near distance.

A few proportions of patients were lost to follow-up at both 1- and 3-month post-operative intervals. At 1 month, 298 questionnaires were distributed, yielding 287 valid responses (96.31%); at 3 months, 122 questionnaires were distributed, yielding 110 valid responses (90.16%). The reasons for high sample dropout rate post-surgery were: 1. Yunnan Province is located in the southwestern border province of China and is home to the largest number of ethnic minorities in China. Its medical level is relatively backward, and hospitals that have the conditions to carry out strabismus surgery are mainly concentrated in the provincial capital city of Kunming. Many strabismus patients come from remote rural areas, and postoperative follow-up is inconvenient, so cooperation is very low. 2. Strabismus surgery usually has a fast recovery, and most patients recover well 1 month after surgery. After that, some patients are not willing to go to the hospital for a follow-up examination again, especially 3 months after surgery. 3. In China, parents attach great importance to their children’s school learning. After the successful surgery, they feel that it will be wasting time to be back to hospital, this is a very common concept in Yunnan, China. Therefore, quite a few number of parents choose to give up follow-up examinations 3 months after surgery. In control group, A total of 385 questionnaires was distributed to healthy children and adolescents, and 342 valid questionnaires were obtained (88.83%).

**FIGURE 1:**
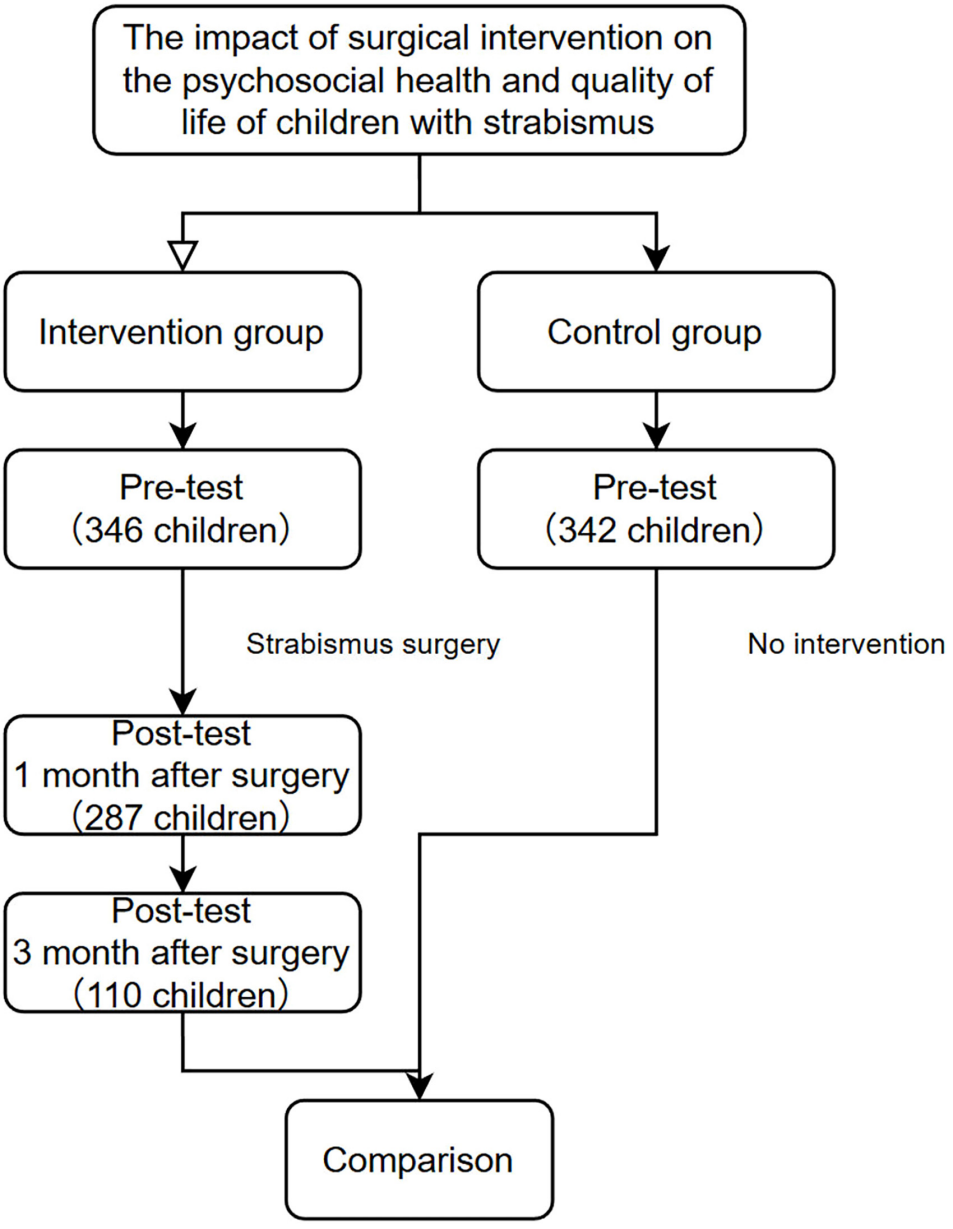
Research methodology flowchart.

Data collected were double-entered using Epidata 3.0, then analyzed and processed with SPSS 23.0 and Jamovi. All tests in this study employed two-tailed hypotheses with a significance level of α = 0.05. The specific experimental methodology is illustrated in Figure 1.

### 2.1 Research tools

The study assessed participants using questionnaires and scales according to three domains: personal baseline data, psychosocial factors (social anxiety, self-esteem, perceived discrimination), and quality of life. The personal baseline data were collected using a self-designed general demographic questionnaire, which included gender, age, ethnicity, residential area, only-child status, school enrollment type, parents’ educational attainment, and monthly household income (see Table 1). Psychosocial factors and quality of life were evaluated using the following four scales.

**TABLE 1:** Descriptive statistics of baseline data.

Variable	Variable classification	Control group (*n* = 342)	Pre-surgery group (*n* = 346)	1-mon post- surgery group (*n* = 287)	3-mon post-surgery group (*n* = 110)	χ^2^ (pre-surgery vs. control group)	Cramer’s V/Phi
Gender	Male	197 (57.6%)	185 (53.5%)	155 (54.0%)	59 (53.6%)	1.190	0.04
	Female	145 (42.4%)	161 (46.5%)	132 (46.0%)	51 (46.4%)		
Age	8–12 (elementary school)	264 (77.2%)	260 (75. 1%)	212 (73.9%)	83 (75.5%)	2.530	0.06
	13–15 (junior high school)	43 (12.6%)	57 (16.5%)	51 (17.8%)	16 (14.5%)		
	16–17 (senior high school)	35 (10.2%)	29 (8.4%)	24 (8.4%)	11 (10.0%)		
Ethnic group	Han ethnic group	267 (78.07%)	252 (72.8%)	204 (71. 1%)	79 (71.8%)	2.546	0.06
	Other ethnic groups	75 (21.93%)	94 (27.2%)	83 (28.9%)	31 (28.2%)		
Place of residence	Rural	132 (38.6%)	150 (43.4%)	128 (44.6%)	52 (47.3%)	1.608	0.05
	Urban	210 (61.4%)	196 (56.6%)	159 (55.4%)	58 (52.7%)		
Live on campus or not	Yes	74 (21.6%)	94 (27.2%)	83 (28.9%)	37 (33.6%)	2.850	0.06
	No	268 (78.4%)	252 (72.8%)	204 (71. 1%)	63 (66.4%)		
Only one child or not	Yes	83 (24.27%)	92 (25.4%)	72 (25. 1%)	27 (24.5%)	0.488	0.03
	No	259 (75.73%)	254 (74.6%)	215 (74.9%)	83 (75.5%)		
Monthly household income (RMB)	3,000–5,000	119 (34.8%)	148 (42.8%)	128 (44.6%)	47 (42.7%)	7.148	0.1
	5,000–8,000	92 (26.9%)	97 (28.0%)	97 (28.0%)	38 (34.5%)		
	8,000–10,000	64 (18.71%)	50 (14.5%)	41 (14.3%)	12 (10.9%)		
	> 10,000	67 (19.59%)	51 (14.7%)	39 (13.6%)	13 (11.8%)		
Mother’s educational attainment	Illiterate/semi-literate	3 (0.01%)	10 (2.9%)	9 (3. 1%)	4 (3.6%)	7.154	0.1
	Elementary school	47 (13.7%)	52 (15.0%)	46 (16.0%)	21 (19. 1%)		
	Junior high school	87 (25.4%)	81 (23.4%)	75 (26. 1%)	21 (19. 1%)		
	High school/technical school/vocational school	63 (18.4%)	79 (22.8%)	61 (21.3%)	27 (24.5%)		
	College degree or above	142 (41.5%)	124 (35.8%)	96 (33.4%)	37 (25.5%)		
Father’s educational attainment	Illiterate/semi-literate	2 (0.01%)	4 (1.2%)	4 (1.4%)	1 (0.9%)	4.206	0.08
	Elementary school	53 (15.5%)	45 (13.0%)	37 (12.9%)	13 (11.8%)		
	Junior high school	92 (20.2%)	109 (31.5%)	101 (35.2%)	41 (37.3%)		
	High school/technical school/vocational school	84 (18.7%)	70 (20.2%)	56 (19.5%)	27 (24.5%)		
	College degree or above	111 (45.0%)	118 (34. 1%)	89 (31.0%)	28 (25.5%)		

*p < 0.05; **p < 0.01; ***p < 0.001.

#### 2.1.1 Child Intermittent Exotropia Questionnaire

Currently, the internationally recognized strabismus assessment scales mainly include AS-20, PedEyeQ, Amblyopia & Strabismus Questionnaire (ASQE), Child Intermittent Exotropia Questionnaire (Child-IXTQ) and so on. AS-20 is primarily applicable to adult strabismus patients, PedEyeQ is a general ophthalmic disease scale that can be rated by parents for younger patients. However, there are few studies by Chinese scholars using this scale as a psychological assessment tool, so its reference value is weak. Although ASQE is frequently used in research on the quality of life in strabismus, of which limitations include being applicable only to children aged 6–12, failing to fully cover infants and older children and adolescents, especially for intermittent exotropia, it is not specifically targeting and may be significant sensitivity (false positives) in the assessment (Felius et al., 2007; Glasman et al., 2013; Hatt et al., 2019; Leske et al., 2021; Wang et al., 2014).

The Child-IXTQ is a specialized questionnaire for assessing the quality of life of children with strabismus, Originally developed by et al. (2010) in the United States, which is good reliability and validity. The questionnaire consists of 12 items and two factors: psychosocial (items 1–8) and visual function (items 9–12). All items use 5-level Likert scoring criteria (100 = never, 75 = rarely, 50 = sometimes, 25 = often, 0 = always). The final score is the average of all items, from which range 0–100. A higher score indicates better quality of life for children with strabismus. Chinese scholars Bian et al. (2015) modified the Child-IXTQ and made it more adapted and validated for Chinese children aged 8–17 years. The Chinese version of the Child-IXTQ demonstrated good reliability and validity, with test-retest reliability coefficients of 0.728–0.913 (*P* < 0.01). In this study, the Cronbach’s α coefficient for the Child-IXTQ scale was 0.856.

Our study included the largest sample size of intermittent exotropia (*n* = 143) and constant exotropia (*n* = 106), both of which belong to different subtypes of the exotropia. Their clinical manifestations and psychological manifestations are similar, and these two sample sizes account for 72%! This is the main reason why we chose the Child-IXTQ scale, as it is more targeted.

#### 2.1 2 Social anxiety scale for children

A scale developed by La Greca for screening social anxiety symptoms in children (La Greca et al., 1988), this scale has good reliability and validity, which is an effective screening tool. Chinese scholars have also revised it (Li et al., 2006) to make it more suitable for Chinese children. The scale consists of 10 items and includes two factors: one is fear of negative evaluation (items 1, 2, 5, 6, 8, and 10), the other is social avoidance and distress (items 3, 4, 7, and 9). This scale uses 3-level scoring criteria (1 = never, 2 = sometimes, 3 = always), with which higher scores indicate greater social anxiety in children. The Cronbach’s α coefficient for the SASC scale in this study was 0.860.

#### 2.1.3 Children’s Self-Esteem Scale

The Children’s Self-Esteem Scale developed by Wei (1997) was used in this study. The scale consists of 26 items and six dimensions: appearance, physical ability, competence, achievement, discipline, public morality and helpfulness. All items use 5-level Likert scoring criteria (1 = strongly disagree, 2 = somewhat disagree, 3 = unsure, 4 = somewhat agree, 5 = strongly agree). Items 3, 4, 10, 17, 20, 22, 23, and 26 are reverse-scored, with which higher scores indicate higher self-esteem levels. The Cronbach’s α coefficient for the CSES in this study was 0.878.

#### 2.1.4 Perceived Discrimination Questionnaire

The Perceived Discrimination Questionnaire developed by Shen (2009), which was used to measure the extent of children’s perceive discrimination at both the individual and group levels, with which each level include three items. All items used 5-level Likert scoring criteria (1 = completely disagree, 2 = somewhat disagree, 3 = unsure, 4 = somewhat agree, 5 = strongly agree), with which higher scores indicate a higher level of perceived discrimination. In this study, the Cronbach’s α coefficients for the total, individual, and group discrimination perception scales was 0.864, 0.759, and 0.858, respectively.

### 2.2 Ethical approval

This study complied with the Declaration of Helsinki. The experimental research protocol, informed consent documents, questionnaire, and psychological evaluation form were reviewed and approved by the Institutional Review Board of the First Affiliated Hospital of Kunming Medical University in Yunnan Province, China. Ethics approval number: (2022) Lunsheng L No. 84.

## 3 Results

### 3.1 Statistical analysis of baseline data

#### 3.1.1 Demographic data of patients with strabismus

Descriptive statistics characterized baseline data. Based on the completeness of the questionnaire survey, the analytical sample included 346 cases before surgery, 287 cases after 1 month surgery followed up, 110 cases after 3 months followed up, and 342 cases without strabismus as controls. The chi-square test revealed no statistical differences between the control group and the preoperative strabismus group in terms of gender, age, ethnicity, residence, mode of education, only one child or not, parental education level, and monthly household income. The effect sizes, Cramer’s V or Phi coefficient, were all ≤ 0.1, indicating weak effects. This suggests that the differences between the two groups are not significant and are comparable (Table 1). Through independent sample *t*-tests and analysis of variance, it was found that demographic variables (gender, age, residence, mode of education, only one child or not, father’s education level, and monthly household income) of strabismus children, as well as the duration and strabismus degree, showed certain differences in quality of life and psychosocial factors (*P* < 0.05). However, there were no differences between ethnic groups (*P* > 0.05).

#### 3.1.2 Sample attrition and baseline comparability analysis

To assess the potential selection bias caused by sample attrition during the follow-up process, this study compared the demographic characteristics and psychosocial indicators between retained participants and attrited participants at two time points: 1 and 3 months after surgery.

#### 3.1.3 Baseline comparison at 1-month postoperative follow-up

Among the 346 participants included at baseline, 59 (17.1%) participants were lost to follow-up 1 month after surgery. The chi-square test and independent sample *t*-test results indicated that there were no statistically significant differences (*p* > 0.05) in almost all baseline variables between the retention group (*n* = 287) and the dropout group (*n* = 59) 1 month later. This suggests that the sample attrition 1 month after surgery may be random and did not introduce systematic bias to the baseline representativeness of the sample. Detailed data are presented in Table 2.

**TABLE 2:** Comparison of baseline at 1-month postoperative follow-up.

Variable		Abscission group (*n* = 59)	Retention group after 1 month (*n* = 287)	χ^2^^/t^	*p*	Cramer’s V /Phi/ *Cohen’s d*
Gender n (%)	Male	31 (52.5%)	154 (53.7%)	0.025	0.876	–0.008
	Female	28 (47.5%)	133 (46.3%)			
Age	8–12 (elementary school)	39 (66.1%)	221 (77.0%)	4.392	0.111	0.120
	13–15 (junior high school)	11 (18.6%)	46 (16.0%)			
	16–17 (senior high school)	9 (15.3%)	20 (7.0%)			
Ethnic group	Han ethnic group	43 (72.9%)	209 (72.8%)	0.000	0.993	0.000
	other ethnic groups	16 (27.1%)	78 (27.2%)			
Place of residence	Rural	26 (44.1%)	124 (43.2%)	0.015	0.903	0.007
	Urban	33 (55.9%)	163 (56.8%)			
Live on campus or not	Yes	23 (39.0%)	71 (24.7%)	5.018	0.025	0.120
	No	36 (61.0%)	216 (75.3%)			
Only one child or not	Yes	16 (27.1%)	76 (26.5%)	0.010	0.920	0.005
	No	43 (72.9%)	211 (73.5%)			
Monthly household income (RMB)	3,000–5,000	29 (49.2%)	119 (41.5%)	2.462	0.482	0.084
	5,000–8,000	14 (23.7%)	83 (28.9%)			
	8,000–10,000	10 (16.9%)	40 (13.9%)			
	>10,000	6 (10.2%)	45 (15.7%)			
Mother’s educational attainment	Illiterate/semi-literate	4 (6.8%)	6 (2.1%)	10.512	0.033	0.182
	Elementary school	14 (23.7%)	38 (13.2%)			
	Junior high school	9 (15.3%)	72 (25.1%)			
	High school/technical school/vocational school	16 (27.1%)	63 (22.0%)			
	College degree or above	16 (27.1%)	108 (37.6%)			
Father’s educational attainment	Illiterate/semi-literate	2 (3.4%)	2 (0.7%)	2.094	0.148	0.126
	Elementary school	7 (11.9%)	38 (13.2%)			
	Junior high school	23 (39.0%)	86 (30.0%)			
	High school/technical school/vocational school	11 (18.6%)	59 (20.6%)			
	College degree or above	16 (27.1%)	102 (35.5%)			
Quality of life (M ± SD)		63.70 ± 30.93	69.03 ± 15.58	–1.289	0.202	–0.224
Psychosocial		64.72 ± 30.81	70.58 ± 16.71	–1.418	0.161	–0.243
Visual function		61.65 ± 35.23	65.92 ± 21.07	–0.898	0.373	–0.150
Social anxiety		15.97 ± 5.41	16.01 ± 3.61	–0.060	0.952	–0.009
Fear of negative evaluation		9.58 ± 3.48	9.73 ± 2.45	–0.327	0.744	–0.051
Social avoidance and distress		6.39 ± 2.21	6.28 ± 1.66	0.366	0.715	0.062
Self-esteem		96.81 ± 22.42	96.75 ± 11.91	0.021	0.983	0.003
Appearance		14.20 ± 4.35	14.08 ± 3.05	0.207	0.836	0.032
Physical education		10.27 ± 3.36	10.03 ± 2.84	0.504	0.616	0.078
Ability		18.58 ± 1.83	18.55 ± 2.34	0.081	0.936	0.014
Sense of accomplishment		14.78 ± 4.12	14.95 ± 2.76	–0.300	0.765	–0.049
Discipline		15.85 ± 4.39	15.58 ± 3.30	0.446	0.657	0.070
Public virtue and helping others		19.92 ± 3.64	20.06 ± 2.85	–0.280	0.780	–0.043
Perception of discrimination		11.66 ± 5.82	11.77 ± 4.92	–0.150	0.881	–0.021
Individual perception of discrimination		6.15 ± 3.12	6.01 ± 2.64	0.327	0.745	0.049
Group perception of discrimination		5.51 ± 3.20	5.76 ± 2.81	–0.611	0.542	–0.084

#### 3.1.4 Baseline comparison of postoperative 3-month follow-up

At 3 months after operation, 110 participants (31.8%) were retained, and the sample loss increased to 236 (68.2%). Baseline comparison showed that there was no significant difference in most demographic and psychosocial variables between the two groups. This indicates that the loss of samples at 3 months after operation is also random, which does not cause systematic bias on the baseline representativeness of the samples. See Table 3 for specific comparison results.

**TABLE 3:** Baseline comparison of postoperative 3-month follow-up.

Variable		Abscission group (*n* = 236)	Retention group after 3-mon (*n* = 110)	χ^2^^/^*^t^*	*p*	Cramer’s V /Phi/ *Cohen’s d*
Gender n (%)	Male	129 (54.7%)	56 (50.9%)	0.425	0.515	0.035
	Female	107 (45.3%)	54 (49.1%)			
Age	8–12 (elementary school)	172 (72.9%)	88 (80.0%)	2.636	0.268	0.087
	13–15 (junior high school)	44 (18.6%)	13 (11.8%)			
	16–17 (senior high school)	20 (8.5%)	9 (8.2%)
Ethnic group	Han ethnic group	165 (69.9%)	87 (79.1%)	3.192	0.074	–0.096
	other ethnic groups	71 (30.1%)	23 (20.9%)			
Place of residence	Rural	91 (38.6%)	59 (53.6%)	6.945	0.008	–0.142
	Urban	145 (61.4%)	51 (46.4%)			
Live on campus or not	Yes	68 (28.8%)	26 (23.6%)	1.016	0.313	0.054
	No	168 (71.2%)	84 (76.4%)			
Only one child or not	Yes	69 (29.2%)	23 (20.9%)	2.666	0.103	0.088
	No	167 (70.8%)	87 (79.1%)			
Monthly household income (RMB)	3,000–5,000	83 (35.2%)	65 (59.1%)	21.211	0.000	0.248
	5,000–8,000	69 (29.2%)	28 (25.5%)			
	8,000–10,000	43 (18.2%)	7 (6.4%)			
	> 10,000	41 (17.4%)	10 (9.1%)			
Mother’s educational attainment	Illiterate/semi-literate	6 (2.5%)	4 (3.6%)	19.220	0.001	0.232
	Elementary school	32 (13.6%)	20 (18.2%)			
	Junior high school	44 (18.6%)	37 (33.6%)			
	High school/technical school/vocational school	53 (22.5%)	26 (23.6%)			
	College degree or above	101 (42.8%)	23 (20.9%)			
Father’s educational attainment	Illiterate/semi-literate	4 (1.7%)	0 (0%)	26.095	0.000	0.263
	Elementary school	27 (11.4%)	18 (16.4%)			
	Junior high school	60 (25.4%)	49 (44.5%)			
	High school/technical school/vocational school	47 (19.9%)	23 (20.9%)			
	College degree or above	98 (41.5%)	20 (18.2%)			
Quality of life (M ± SD)		68.79 ± 21.76	66.67 ± 11.59	1.184	0.237	0.110
Psychosocial		69.94 ± 22.37	68.81 ± 13.19	0.590	0.556	0.056
Visual function		66.50 ± 25.78	62.39 ± 19.71	1.632	0.104	0.169
Social anxiety		16.10 ± 4.29	15.79 ± 3.16	0.756	0.450	0.077
Fear of negative evaluation		9.76 ± 2.80	9.59 ± 2.29	0.590	0.556	0.063
Social avoidance and distress		6.34 ± 1.89	6.20 ± 1.44	0.777	0.438	0.079
Self-esteem		96.76 ± 15.77	96.75 ± 10.17	0.006	0.995	0.001
Appearance		14.06 ± 3.50	14.18 ± 2.83	-0.335	0.738	-0.036
Physical education		10.04 ± 3.15	10.15 ± 2.42	-0.377	0.706	–0.037
Ability		18.53 ± 2.21	18.63 ± 2.37	–0.390	0.697	–0.044
Sense of accomplishment		14.85 ± 3.18	15.07 ± 2.68	–0.644	0.520	–0.072
Discipline		15.75 ± 3.72	15.35 ± 2.99	1.094	0.275	0.113
Public virtue and helping others		19.88 ± 3.16	20.35 ± 2.57	–1.479	0.140	–0.156
Perception of discrimination		12.00 ± 5.24	11.21 ± 4.68	1.416	0.158	0.154
Individual perception of discrimination		6.18 ± 2.82	5.73 ± 2.51	1.496	0.136	0.163
Group perception of discrimination		5.83 ± 2.92	5.48 ± 2.76	1.038	0.300	0.120

### 3.2 Comparison of scores for psychosocial and quality of life between strabismus patients and healthy children

Independent samples *t*-tests were performed on the scores for quality of life, self-esteem, social anxiety, perceived discrimination and all of their subdimensions between the pre-surgery group and the control group. The control group revealed significantly higher scores on quality of life, self-esteem, and their subdimensions (*p* < 0.001) compared to the pre-surgery strabismus group. Conversely, the control group had significantly lower scores in social anxiety, perceived discrimination, and their subdimensions compared to the pre-surgery group (*p* < 0.001). The effect size was calculated, and Cohen’s *d* > 0.5, indicating a medium-to-large effect (Table 4).

**TABLE 4:** Differences in variables and subdimensions between the control group and the pre-surgery group.

Variable	Control group (*n* = 342) *M ± SD*	Strabismus group (*n* = 346) *M ± SD*	*t*	Cohen’s *d*
Quality of life	87. 18 ± 6.06	68. 11 ± 19.13	–17.572***	1.16
Psychosocial	86.65 ± 6.75	69.58 ± 19.90	–15.031***	1.07
Visual function	88.23 ± 11.10	65. 19 ± 24.07	–16.094***	1.11
Self-esteem	105. 10 ± 7.31	96.76 ± 14.22	–9.662***	0.86
Appearance	15.96 ± 2.15	14. 10 ± 3.30	–8.773***	0.82
Physical education	11.36 ± 2.19	10.08 ± 2.93	–6.838***	0.70
Ability	23.44 ± 3.08	18.56 ± 2.26	–23.729***	1.35
Sense of accomplishment	15.86 ± 2.13	14.92 ± 3.03	–4.711***	0.60
Discipline	16.96 ± 1.94	15.62 ± 3.50	–6.203***	0.69
Public virtue and helping others	21.61 ± 1.96	20.03 ± 2.99	–8.170***	0.79
Social anxiety	14.66 ± 2.21	16.00 ± 3.96	5.486***	0.65
Fear of negative evaluation	9.04 ± 1.66	9.71 ± 2.64	3.977***	0.55
Social avoidance and distress	5.66 ± 1. 14	6.30 ± 1.76	5.657***	0.66
Perception of discrimination	9.58 ± 2.20	11.75 ± 5.07	7.281***	0.74
Individual perception of discrimination	4.55 ± 1.44	6.03 ± 2.73	8.940***	0.82
Group perception of discrimination	5.03 ± 1.47	5.72 ± 2.87	3.940***	0.55

***p < 0.001.

### 3.3 Differences on psychosocial and quality of life among the four strabismus types

Patients with strabismus in the study were classified into constant exotropia, esotropia, intermittent exotropia and vertical strabismus. ANOVA revealed significant differences among the four strabismus types in quality of life, psychosocial scores, social anxiety, and its subdomains (fear of negative evaluation; social avoidance and distress). Further LSD testing demonstrated that intermittent exotropia had significantly higher quality of life (72.16 ± 18.11) and psychosocial (73.73 ± 18.38) scores than constant exotropia, esotropia, and vertical strabismus, however, there was no significant differences among the latter three types. The vertical strabismus group had the highest scores on the total social anxiety score and the two sub-dimensions (18.52 ± 5.03, 10.97 ± 3.36, and 7.55 ± 2.01, respectively), which were significantly higher than those of the other three groups, while there was no statistical difference among these three groups. There was no statistical difference in the scores of these four strabismus groups on self-esteem with sub-dimensions, discrimination perception with sub-dimensions. Calculating the partial η^2^ of the effect size, we found that the effect sizes of variables with significant differences ranged from 0.03 to 0.06, indicating a generally moderate effect (Table 5).

**TABLE 5:** Difference test of variables and sub-dimensions in strabismus classification.

Variable	➀Constant exotropia (*n* = 106) *M ± SD*	➁Esotropia (*n* = 66) M ± SD	➂Intermittent exotropia (*n* = 143) *M ± SD*	➃Vertical strabismus (*n* = 31) *M ± SD*	*F*	*LSD*	*η^2^_p_*
Quality of life	66.35 ± 19. 12	63.92 ± 19.55	72.16 ± 18. 11	64.45 ± 20.21	3.966**	➂>➀➁➃	0.034
Psychosocial	68.60 ± 20.21	64.63 ± 19.85	73.73 ± 18.38	64.31 ± 22.43	4.370**	➂>➀➁➃	0.037
Visual function	61.85 ± 25. 12	62.50 ± 25.00	69.01 ± 23.38	64.72 ± 19.60	2.184	➂>➀	0.019
Social anxiety	16.08 ± 3.67	15.79 ± 4.05	15.50 ± 3.71	18.52 ± 5.03	5.192**	➃>➀➁➂	0.044
Fear of negative evaluation	9.92 ± 2.52	9.50 ± 2.64	9.37 ± 2.49	10.97 ± 3.36	3.552*	➃>➀➁➂	0.030
Social avoidance and distress	6. 17 ± 1.64	6.29 ± 1.90	6. 13 ± 1.63	7.55 ± 2.01	6.123***	➃>➀➁➂	0.051
Self-esteem	96.04 ± 15.04	96.89 ± 13.31	98.06 ± 13.66	92.94 ± 15.48	1.244		0.011
Appearance	13.71 ± 3.41	14.32 ± 3.02	14.40 ± 3.20	13.61 ± 3.87	1.215		0.011
Physical education	9.98 ± 2.86	9.61 ± 3.09	10.50 ± 2.89	9.42 ± 2.87	2.154	➂>➁	0.019
Ability	18.32 ± 1.74	18.77 ± 2.62	18.57 ± 2.31	18.87 ± 2.72	0.787		0.007
Sense of accomplishment	14.80 ± 3.24	14.91 ± 2.90	15. 13 ± 2.93	14.35 ± 3.03	0.649		0.006
Discipline	15.65 ± 3.51	15.26 ± 3.93	15.86 ± 3.41	15.23 ± 2.97	0.591		0.005
Public virtue and helping others	19.90 ± 2.79	20.30 ± 3.16	20.06 ± 3.07	19.77 ± 3.02	0.333		0.003
Perception of discrimination	11.76 ± 5.24	11.92 ± 4.96	11.40 ± 4.78	12.97 ± 6.01	0.849		0.007
Individual perception of discrimination	6. 15 ± 2.74	5.79 ± 2.66	5.95 ± 2.67	6.55 ± 3. 11	0.654		0.006
Group perception of discrimination	5.61 ± 2.98	6.14 ± 2.89	2.45 ± 2.64	6.42 ± 3. 38	1.558		0.013

*p < 0.05;

**p < 0.01;

***p < 0.001.

### 3.4 Differences in prism diopters of strabismus in variables and subdimensions

Exotropia is denoted by a “-” mark, esotropia is denoted by a “+” mark, and prism diopters of strabismus is denoted by “Δ”mark. The same as below. We compared the impact of mild strabismus ( < 30Δ) and severe strabismus ( ≥ 30Δ) on quality of life and psychosocial factors. For exotropia, the mild group (-30Δ < ∼ ≤ -15Δ) showed significantly higher quality of life, self-esteem with its subdimensions scores (appearance, public morality, and helping others) than the severe group ( ≤ -30Δ) (*p* < 0.05); but showed lower scores on the social anxiety subdimension (social avoidance and distress) and individual discrimination perception scores than the severe group ( ≤ -30Δ) (*p* < 0.05) (Table 6).

**TABLE 6:** Difference prims diopters of the exotropia in various variables with subdimensions.

Variable	-15^Δ^≤ < -30^Δ^ (*n* = 107)	≤ -30^Δ^ (*n* = 142)	*t*	Cohen’s *d*
Quality of life	73.59 ± 15.51	65.63 ± 18.02	4.490***	0.685
Psychosocial	77.16 ± 15.14	67.12 ± 19.94	4.517***	0.746
Visual function	71.85 ± 22.87	62.63 ± 21.87	3.227***	0.643
Social anxiety	15.55 ± 3.64	16.49 ± 3.86	**–**1.954	0.500
Fear of negative evaluation	9.53 ± 2.47	9.98 ± 2.61	**–**1.368	1.652
Social avoidance and distress	6.02 ± 1.55	6.51 ± 1.77	**–**2.303*	0.540
Self-esteem	100.22 ± 14.24	93.80 ± 12.66	3.759***	0.693
Appearance	15.07 ± 3.17	13.41 ± 3.11	4.131***	0.728
Physical education	10.50 ± 2.78	9.86 ± 2.93	1.758	0.473
Ability	18.63 ± 2.23	18.38 ± 2.01	0.911	0.344
Sense of accomplishment	15.21 ± 2.92	14.58 ± 2.96	1.672	0.463
Discipline	16.12 ± 3.18	15.29 ± 3.55	1.917	0.494
Public virtue and helping others	20.47 ± 2.77	19.46 ± 2.95	2.743**	0.593
Perception of discrimination	11.13 ± 4.72	12.24 ± 5.04	**–**1.765	0.476
Individual perception of discrimination	5.71 ± 2.48	6.46 ± 2.76	**–**2.208*	0.533
Group perception of discrimination	5.42 ± 2.72	5.78 ± 2.81	**–**1.019	0.360

*p < 0.05;

**p < 0.01;

***p < 0.001.

For esotropia, the mild group (+10Δ ≤ ∼ < +30Δ) showed higher scores for quality of life, self-esteem with its subdimensions (appearance, sense of achievement, public morality, and helping others) than the severe group ( ≥ +30Δ), but showed lower scores for discrimination perception. The rest of the indicators were not affected by the prims diopters of strabismus (*p* > 0.05) (Table 7). This result show that the prims diopters of the exotropia and esotropia may influence the quality of life and social psychological factors.

**TABLE 7:** Difference prims diopters of the esotropia in various variables with subdimensions.

Variable	+10^Δ^≤ < +30^Δ^ (*n* = 24)	>+30^Δ^ (*n* = 42)	*t*	Cohen’s *d*
Quality of life	76.04 ± 15.43	61.31 ± 15.15	3.775***	0.983
Psychosocial	77.86 ± 13.94	62.43 ± 15.27	4.075***	1.021
Visual function	72.40 ± 27.02	59.08 ± 20.11	2.279*	0.764
Social anxiety	15.13 ± 3.22	15.90 ± 4.13	–0.796	0.448
Fear of negative evaluation	9.13 ± 2.47	9.55 ± 2.62	–0.644	0.404
Social avoidance and distress	6.00 ± 1.32	6.36 ± 2.00	–0.873	0.449
Self-esteem	102.46 ± 13.66	92.86 ± 11.54	3.039**	0.882
Appearance	15.38 ± 2.45	13.50 ± 3.19	2.490*	0.799
Physical education	10.00 ± 3.30	9.43 ± 2.54	0.788	0.448
Ability	19.46 ± 2.67	18.31 ± 3.01	1.553	0.631
Sense of accomplishment	16.50 ± 2.78	14.12 ± 2.63	3.462***	0.942
Discipline	15.83 ± 3.28	15.07 ± 3.70	0.838	0.462
Public virtue and helping others	21.54 ± 2.43	19.24 ± 3.29	2.992**	0.874
Perception of discrimination	10.83 ± 4.50	12.29 ± 5.59	–1.087	0.529
Individual perception of discrimination	5.75 ± 2.72	5.67 ± 2.98	0.113	0.166
Group perception of discrimination	5.08 ± 2.41	6.62 ± 3.07	–2.106*	1.590

*p < 0.05;

**p < 0.01;

***p < 0.001.

Compared to the small prim diopters of the strabismus, the big ones may reduce the quality of life and self-esteem scores, increase the social anxiety and perceived discrimination scores. There is a little difference between the esotropia and exotropia. Through the calculation of the effect size Cohen’s *d*, variables with significant differences all have values above 0.5, indicating a moderate or above effect.

### 3.5 Analysis of differences on psychosocial and quality of life with strabismus children pre and post-surgery

The data of strabismus patients before operation, 1 and 3 months after operation were analyzed by repeated measurement ANOVA. The results showed that the overall score and sub-dimension scores of patients’ quality of life and self-esteem were generally higher than those before operation. The score had an increasing trend 1 month after operation, but there was no significant difference. The score increased significantly 3 months after operation (*P* = 0.000). The ability sub-dimension of self-esteem was significantly improved at 1 and 3 months after operation [*f*(2,218) = 106.440, *P* = 0.000]; The sports sub-dimension of self-esteem decreased slightly at 1 month after operation, but increased significantly at the second follow-up at 3 months after operation [*f*(2,218) = 6.325, *P* = 0.002]; However, there was no significant difference in these 3 sub-dimensions of self-esteem: sense of achievement, discipline, merit and helping others. There was no significant difference in social anxiety and sub-dimension scores between the two groups at 1 month after operation, but it was significantly lower at 3 months after operation [*f*(1.878204.688) = 42.467, *P* = 0.000]. There was a certain difference in patients’ perception of discrimination after operation and before operation, but it did not reach the traditional statistical significance level (*P* = 0.056). The perception of individual discrimination decreased significantly at 1 and 3 months after operation [*f*(2,218) = 4.754, *P* = 0.010], and the perception of group discrimination decreased significantly at 3 months after operation [*f*(2,218) = 3.084, *P* = 0.048]. In this study, the effect bias η 2 of repeated measurement analysis of variance was calculated, and the results showed that both of them had medium and high response (see Table 8 for details).

**TABLE 8:** Analysis of variance of repeated measurement of social psychological factors.

Variable	➀Pre-operation (*n* = 110) *M ± SD*	➁ 1-mon post-surgery (*n* = 110) *M ± SD*	➂ 3-mon post-surgery (*n* = 110) *M ± SD*	*F*	Partial η^2^_p_	Post comparison
Quality of life	66.67 ± 11.59	68.81 ± 10.80	81.72 ± 11.11	92.377***	0.459	➂>➀,➂>➁
Psychosocial	68.81 ± 13.19	70.65 ± 12.94	83.15 ± 12.03	61.410***	0.360	➂>➀,➂>➁
Visual function	62.39 ± 19.71	65.11 ± 16.60	78.86 ± 15.52	35.121***	0.244	➂>➀,➂>➁
Social anxiety	15.79 ± 3.16	16.16 ± 3.27	12.85 ± 2.54	42.467***	0.280	➁>➂,➀>➂
Fear of negative evaluation	9.59 ± 2.29	9.88 ± 2.35	7.81 ± 1.84	31.979***	0.227	➁>➂,➀>➂
Social avoidance and distress	6.20 ± 1.44	6.28 ± 1.35	5.04 ± 1.09	30.581***	0.219	➁>➂,➀>➂
Self-esteem	96.75 ± 10.17	97.16 ± 10.46	102.45 ± 13.05	11.257***	0.094	➂>➀,➂>➁
Appearance	14.18 ± 2.83	14.54 ± 2.86	15.58 ± 2.44	8.626***	0.073	➂>➀,➂>➁
Physical education	10.15 ± 2.42	9.85 ± 2.64	10. 96 ± 2.21	6.325***	0.055	➂>➀,➂>➁
Ability	18.63 ± 2.37	22.04 ± 4.14	24.88 ± 3.75	106.440***	0.494	➂>➀,➂>➁,➁>➀
Sense of accomplishment	15.07 ± 2.68	14.88 ± 2.37	15. 13 ± 3.34	0.238	0.002	
Discipline	15.35 ± 2.99	15.89 ± 2.67	16.28 ± 2.94	2.998	0.027	
Public virtue and helping others	20.35 ± 2.57	19.97 ± 2.62	19.62 ± 3.97	1.668	0.015	
Perception of discrimination	11.21 ± 4.68	10.77 ± 4.13	9.98 ± 4.01	2.923	0.026	
Individual perception of discrimination	5.73 ± 2.51	4.99 ± 2.17	4.96 ± 2.15	4.754**	0.042	➀>➁,➀>➂
Group perception of discrimination	5.48 ± 2.76	5.78 ± 2.43	5.02 ± 2.31	3.084*	0.028	➁>➂

The *F*-value degree of freedom has been corrected by Greenhouse-Geisser according to the spherical test results.

*p < 0.05;

**p < 0.01;

****p < 0.001.

## 4 Discussion

### 4.1 Children with strabismus have poorer mental health and quality of life than healthy children

This study found that children with strabismus have lower self-esteem and quality of life, higher levels of perceived discrimination and social anxiety compared to healthy peers of the same age. Overall, their mental health and quality of life are worse. This may be due to the fact that the appearance of strabismus can have negative impacts on interpersonal relationships. People with strabismus are often viewed as different by those around them and are subject to discrimination and exclusion. As a result, they may experience negative psychological emotions such as inferiority, anxiety, depression, and loneliness. In addition, strabismus in childhood can affect visual development, leading to amblyopia and stereoscopic vision disorders, which can result in decreased learning and motor abilities and affecting daily life (e.g., falling down stairs). This may aggravate the anxiety of strabismus patients, and their self-esteem is easily frustrated. This study found that patients with strabismus had lower self-esteem and quality of life scores than normal people, while their scores for social anxiety and perceived discrimination were higher. This is consistent with the results of low self-esteem, social phobia, depression and anxiety, and low quality of life of strabismus patients found by some scholars (Buffenn, 2021; Gouveia-Moraes et al., 2023; Schuster et al., 2019; Silva et al., 2022).

Children and adolescents aged 8–17 are in a critical period of character formation and physical and mental development. The negative effects of strabismus stem from poor visual development on the one hand and facial defects on the other, both of which are detrimental to children’s psychological development and increase the risk of psychological or mental illness. Therefore, it is essential to detect strabismus early and treat it surgically as soon as possible. This not only promotes visual and visual function development of children with strabismus but also helps to reverse the negative psychological effects caused by the appearance of strabismus, thereby improving the quality of life for patients with strabismus.

### 4.2 The impact of strabismus type and severity on mental health and quality of life

Some scholars have shown that esotropia has a greater impact on interpersonal relationships than exotropia, especially in case of big angles esotropia, which tends to receive more negative evaluations (Goff et al., 2006; Olitsky et al., 1999). While studies on vertical strabismus are less common. The results of this study differ from those of previous studies. We compared patients with strabismus into four categories: constant exotropia, esotropia, intermittent exotropia, and vertical strabismus. This study found that among these four types of strabismus, intermittent exotropia has better mental health and quality of life than other types of strabismus. Intermittent exotropia scored highest on the quality-of-life scale, while vertical strabismus scored lowest on the social anxiety scale. It is possible to analyze that intermittent exotropia is a type of mild strabismus, which can self-control the normal position. Exotropia only appears when looking at the distance or distracting. The appearance of strabismus is not a permanent condition and there is no different from normal people, so the impact of appearance is less than of other types of strabismus. Furthermore, the likelihood of amblyopia developing in intermittent exotropia is lower. Patients typically have some stereoscopic vision and relatively good visual function, which means that the impact on daily activities is less than with other types of strabismus. Therefore, patients with intermittent exotropia tend to have better mental health and quality of life than those with other types of strabismus. Vertical strabismus, the appearance is characterized by uneven eye height, tilted neck and asymmetrical facial development. Some cases even involve scoliosis. Visual symptoms include double vision, amblyopia, lack of stereoscopic vision, and refractive errors. Vertical strabismus has the worst visual function and appearance defects among the four types of strabismus, and is much more likely to cause anxiety and inferiority in patient. This is consistent with the results of our study, which showed that vertical strabismus had the highest social anxiety score and a relatively low quality of life score. In addition, most types of strabismus patients are more sensitive and have low self-esteem. Their self-esteem is easily frustrated and they are easy to perceive discrimination from individuals or groups. The results of this study found that there was no significant difference in the scores of self-esteem, discrimination and its sub-dimensions.

The study found that the degree of strabismus also has a great correlation with social psychological factors. The more severe the strabismus, the more obvious the appearance defects will be; at the same time, the greater the impact on visual function and mental health. For example, Uretmen et al. (2003) found that children with severe strabismus are more susceptible to discrimination, particularly with esotropia causing greater harm to self-esteem. We also found that the degree of strabismus was correlated with quality of life and social psychological factors. Patients with mild strabismus (below 30Δ) scored higher in quality of life and partial self-esteem, and lower in social anxiety and perceived discrimination compared to those with severe strabismus (above 30Δ). There are certain differences in the psychological impact of esotropia and exotropia. These differences may be caused by the appearance and visual function between the two types of strabismus: exotropia gives people the impression that the person is not looking at them directly and is disrespectful, while esotropia gives people the impression that the person is selfish and narrow-minded. In terms of visual function, exotropia does not easily cause amblyopia, but it lacks stereopsis; while esotropia easily causes amblyopia, but has partial stereopsis. As the angle of strabismus increases, patients are more likely to experience significant social anxiety and feeling of discrimination, which can reduce their quality of life and affect the normal social interactions in daily life.

### 4.3 The impact of strabismus surgery on mental health and quality of life

Previous studies in other countries have shown that surgery has positive effects on the mental health and quality of life for patients with strabismus (Archer et al., 2005). However, there is a lack of relevant research in China. For example, Ehlers et al. (2023) found through a questionnaire survey that patients’ quality of life related to strabismus improved 3 months after strabismus surgery, while anxiety and depression levels decreased. Menon et al. (2002) surveyed 40 strabismus patients aged 15–25 3 months after surgery and found that 95% of patients perceived a significant improvement in their self-confidence and self-esteem. Xu et al. (2012) also found that Chinese adolescents and adults with strabismus experienced significant improvements in self-esteem, self-confidence, and interpersonal relationships 2–3 months after undergoing strabismus correction surgery. Strabismus correction surgery primarily serves an aesthetic purpose. Improving the appearance of strabismus can prevent others from forming preconceived stereotypes and negative evaluations, thereby reducing the perception of discrimination among strabismus patients. Secondly, early surgery for children with strabismus can prevent the occurrence of strabismic amblyopia and promote the formation of normal stereoscopic vision, which is beneficial to improving their quality of life. Mojon-Azzi et al. (2011) believe that negative attitudes toward strabismus patients among peers appear around the age of 6, so having strabismus surgery before the age of 6 is more beneficial to their physical and mental health development. Another study even found that changes in the quality of life of children with strabismus can also promote changes in the quality of life of their parents (Temeltürk et al., 2022).

This study shows that strabismus surgery has very positive effects on changes in quality of life and mental health, but there is a certain degree of delay. According to the statistical results, the quality of life and self-esteem were not significantly improved 1 month after operation, and even the score of physical exercise was decreased, but the quality of life and self-esteem were significantly improved 3 months after operation. Social anxiety did not change significantly at 1 month after operation, but improved significantly at 3 months after operation. The perception of discrimination was improved after operation, but it did not reach the traditional level of statistical significance. However, the sub-dimension scores of individual discrimination perception were significantly improved at 1 and 3 months after operation, while the group discrimination perception scores were improved at 3 months. We believe that after strabismus surgery for a period of time, mainly 3 months, the positive self-esteem is increasing, the negative anxiety and discrimination perception are decreasing, and the quality of life has been significantly improved. The change was not obvious at 1 month, which is a major feature of this study different from other studies. The effect of surgery on self-esteem was also limited, and there was no significant change in the three dimensions of sense of achievement, discipline, merit and help. The surgery improved patients’ appearance and visual function, enhanced their social adaptation abilities and quality of life, and helped restore their confidence and self-esteem.

Strabismus is a disease involving ophthalmology and psychology. From an ophthalmological perspective, the effectiveness of treatment is primarily assessed based on the recovery of eye position and visual function. However, from a psychological perspective, assessing the effectiveness of treatment also requires evaluating changes in patients’ mental health and quality of life. Hatt et al. (2012) study found that 95% of strabismus patients achieved successful surgeries based on traditional surgical criteria, but when postoperative quality of life improvement was added into the criteria for successful treatment, this proportion dropped to 50%. This explains why many strabismus patients, even after successful surgery, still stubbornly believe that their eyes remained in a strabismus state and have not been completely cured. They still experience feelings of discrimination and a lack of respect from others. Therefore, surgery is a primary treatment for strabismus, and psychological intervention can further improve the effectiveness of treatment. According to the results of this study, psychological intervention is recommended to be implemented about 1 month after strabismus surgery, which is more conducive to improving the mental health level of strabismus patients. In addition, it is essential to quantitatively assess the mental health and quality of life of strabismus patients before and after surgery, as this can serve as a basis for psychological intervention and counseling for strabismus.

This study involves ophthalmology and psychology, examining the dynamic changes in quality of life and psychosocial factors in patients 1 and 3 months after strabismus surgery with a large sample size. Compared to previous studies that only classified strabismus into exotropia and esotropia, this study provides a more detailed classification of strabismus types and degrees. Focusing on the less being explored area of discrimination perception in children with strabismus, this study reflects the psychological and personality characteristics of strabismic children in ethnic minority areas in Southwest China. Currently, research on strabismus related psychology in China is relatively scarce compared to other developed countries worldwide, especially in ethnic minority areas in Southwest China, where such research is still lacking. The results of this study also fill this gap.

## Data Availability

The raw data supporting the conclusions of this article will be made available by the authors, without undue reservation.
